# EGFR inhibition attenuates diabetic nephropathy through decreasing ROS and endoplasmic reticulum stress

**DOI:** 10.18632/oncotarget.15948

**Published:** 2017-03-06

**Authors:** Zheng Xu, Yunjie Zhao, Peng Zhong, Jingying Wang, Qiaoyou Weng, Yuanyuan Qian, Jibo Han, Chunpeng Zou, Guang Liang

**Affiliations:** ^1^ Chemical Biology Research Center, School of Pharmaceutical Sciences, Wenzhou Medical University, Wenzhou, Zhejiang, 325035, China; ^2^ Department of Ultrasonography, the Second Affiliated Hospital and Yuying Children's Hospital of Wenzhou Medical University, Wenzhou, Zhejiang, 325000, China; ^3^ Department of Interventional Radiology, the Fifth Affiliated Hospital of Wenzhou Medical University, Lishui, Zhejiang, 323000, China

**Keywords:** diabetic nephropathy, epidermal growth factor receptor, inhibitor, ER stress, oxidative stress

## Abstract

Diabetic nephropathy (DN) is a progressive kidney disease due to glomerular capillary damage in diabetic patients. Endoplasmic reticulum (ER) stress caused by reactive oxygen species (ROS) is associated with DN progression. Epidermal growth factor receptor (EGFR) mediates oxidative stress and damage of cardiomyocytes in diabetic mice. Here we demonstrated that AG1478, a specific inhibitor of EGFR, blocked EGFR and AKT phosphorylation in diabetic mice. Oxidative stress and ER stress markers were eliminated after AG1478 administration. AG1478 decreased pro-fibrotic genes TGF-β and collagen IV. Furthermore, we found that high glucose (HG) induced oxidative stress and ER stress, and subsequently increased ATF4 and CHOP. These changes were eliminated by either AG1478 or ROS scavenger N-acetyl-L-cysteine (NAC) administration. These results were confirmed by knock-down approaches in renal mesangial SV40 cells. However, AG1478, not NAC, reversed HG induced EGFR and AKT phosphorylation. These results suggest that EGFR/AKT/ROS/ER stress signaling plays an essential role in DN development and inhibiting EGFR may serve as a potential therapeutic strategy in diabetic kidney diseases.

## INTRODUCTION

Diabetes mellitus is a major health risk worldwide. The metabolic abnormality and microvascular complications caused by the sustained hyperglycemia lead to diabetic nephropathy (DN) [1]. Diabetic kidney disease is the leading cause of morbidity and end-stage renal disease (ESRD) [2–4]. However, effective clinical interventions for DN have not yet been elucidated. Although great advances have been accomplished in tight glycemic control and blood pressure control [5, 6], they cannot stop DN progression.

Many receptor tyrosine kinases (RTKs) are implicated in DN pathogenesis. The epidermal growth factor receptor (EGFR) is an important member of RTKs [7]. After ligand binding, EGFR signaling is triggered by phosphorylation cascades intracellularly. EGFR is widely expressed in glomeruli, proximal tubes and collecting ducts. EGFR phosphorylation is increased in diabetic animals [8], suggesting its activation is involved in diabetic damages. Furthermore, recent studies have demonstrated that pharmacological antagonists of EGFR efficiently block downstream cascades activation in diabetic animals [8]. EGFR is pathogenic in various nephron cell types. Hyperglycemia increases reactive oxygen species (ROS) in diabetic patients. Accumulating evidence indicates that the ROS overproduction contributes to DN pathogenesis. We previously demonstrated that activation of EGFR leads to cardiac damage and remodeling via ROS generation in STZ-induced diabetic mice [9]. Inhibiting EGFR protects cardiomyocytes from oxidative stress by reducing ROS accumulation. Blockage of AKT prevents HG-induced ROS generation and cardiomyocyte damage [9]. Thus, inhibiting EGFR activation may also serve as a therapeutic target for DN. In addition, Endoplasmic reticulum (ER) stress, with misfolded and/or unfolded proteins in ER membranes, has been shown to mediate DN development and progression [10]. The crosstalk between ER stress and ROS production is well established in cancer biology, in which increasing ROS induces ER stress to trigger cancer cell apoptosis [11–13]. Interestingly, ERGR signaling plays a key role regulating ER stress and ROS accumulation [14, 15]. However, whether EGFR-mediated ER stress and ROS generation contribute to DN progression remains unexplored. Although several mechanisms are involved in the regulation of EGFR, the interactions between EGFR, ROS generation/ER stress are unknown in the progression of DN.

In this study, we investigated the role of EGFR/AKT/ROS/ER stress pathway in the progression of DN using AG1478, a specific and commercial inhibitor of EGFR. AG1478 inhibited EGFR and AKT activation, ameliorating ROS accumulation and ER stress and preventing diabetes-induced renal injury. Thus blocking EGFR/AKT/ROS/ER stress pathway may serve as a potential therapeutic strategy in preventing diabetic nephropathy.

## RESULTS

### AG1478 administration decreased diabetes-induced renal injury

**Figure 1:**
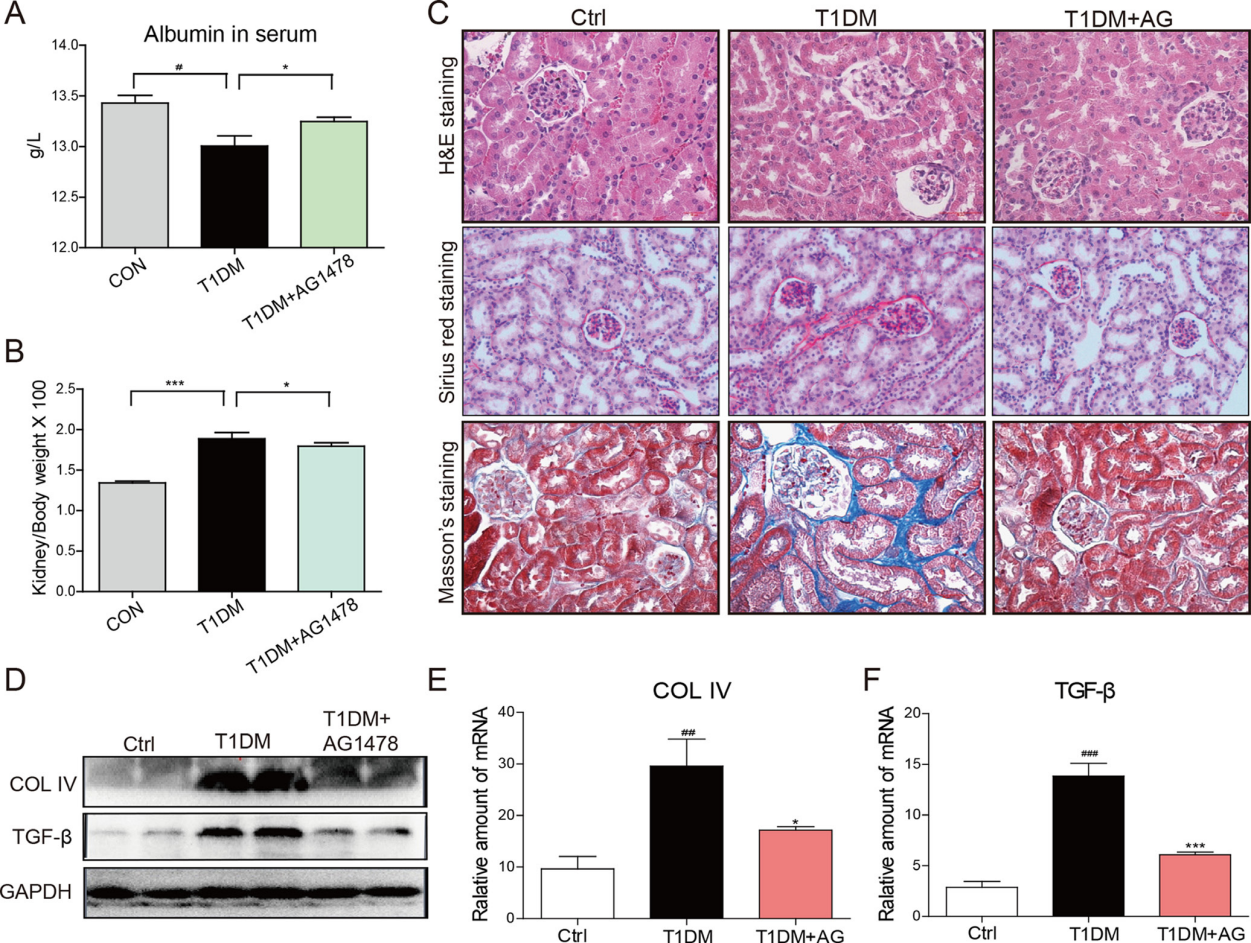
AG1478 attenuate diabetes-induced renal histological abnormalities and fibrosis The effects of AG1478 on the metabolic profiles of STZ-induced mice: serum albumin (**A**) and kidney/body weight ratio (**B**) Serum albumin and kidney/body weight ratio were detected at the time of death. Data are mean ± S.E.M. (**C**) Administration of AG1478 significantly improved histologic abnormalities and fibrosis in the formalin-fixed renal tissues. Renal histopathologic analysis was performed using H&E staining (400×); representative figures of Sirius red staining and Masson trichrome staining (400×) on renal tissue. (**D**) Western blot analysis for Col4 and TGF-β1 protein expression in the renal tissue. (**E** and **F**) The mRNA expression of the Col4 and TGF-β1 in the renal tissues was measured by real-time qPCR. (Eight mice in each group were used for above analysis; *P < 0.05, ***P < 0.001 versus DN; ^#^*P* < 0.05, ^##^*P* < 0.01, ^###^*P* < 0.001 versus vehicle control (Ctrl)).

**Figure 2:**
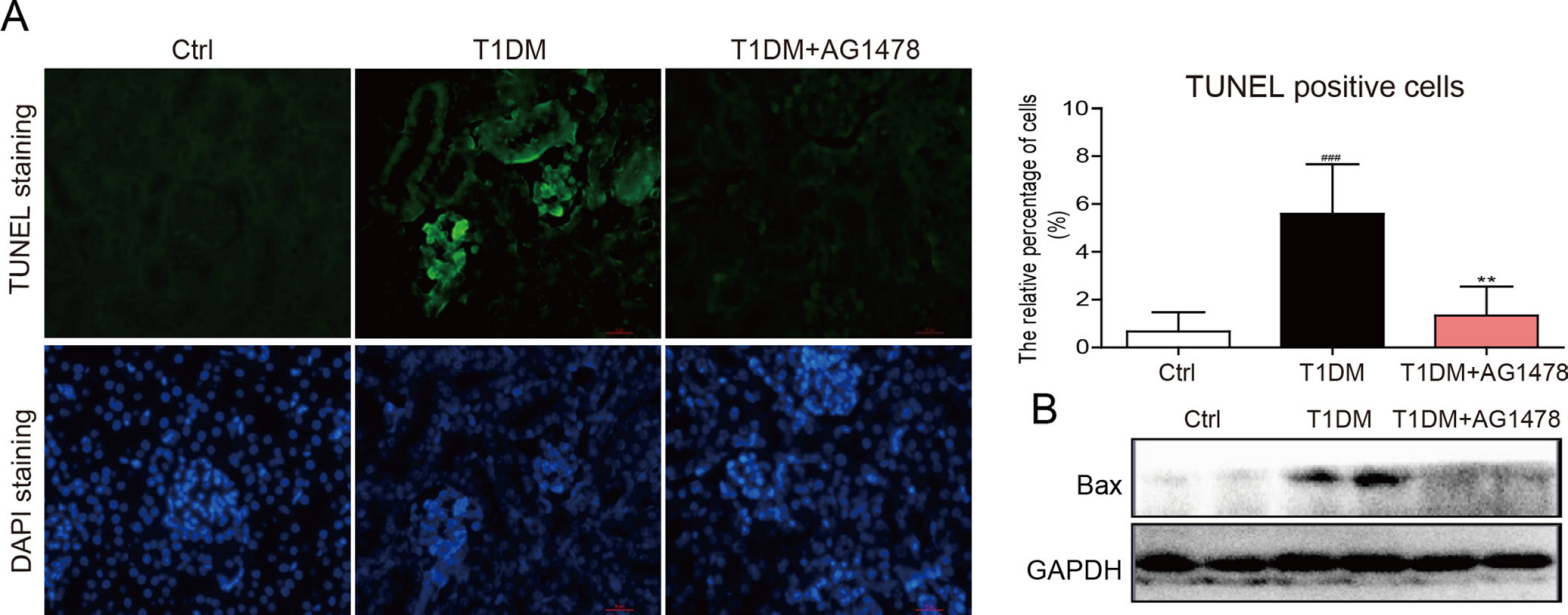
AG1478 mitigate apoptosis in diabetic kidney (**A**) Representative images for TUNEL staining in renal tissue sections. Statistic data of TUNEL positive cell was shown, data were presented as mean ± SDs; (**B**) Western blot analysis for the protein expression of apoptosis-related proteins Bax in renal tissues. (Eight mice in each group were used for above analysis. **P < 0.01 versus DN; ^###^*P* < 0.001 versus vehicle control (Ctrl)).

Streptozotocin (STZ)-induced diabetic mice showed elevated blood glucose level (Supplementary Figure 1A) and the relatively lower body weight (Supplementary Figure 1B). However, AG1478 (20mg/kg) treatment for 8 weeks did not affect blood glucose level or body weight in either T1DM group or AG1478-treated group (Supplementary Figure 1). Renal injury was examined by albumin reagent kit, and renal fibrosis was examined by Sirius Red staining and Masson's trichrome staining, respectively. The diabetic animals induced by STZ injection showed decreased serum albumin, elevated kidney/body weight ratio, histological abnormalities, and renal fibrosis (Figure 1A–1C). Administration of AG1478 significantly improved serum albumin level and decreased the kidney/body weight ratio in diabetic mice (Figure 1A and 1B). Histological abnormalities and fibrosis in diabetic mice were ameliorated by AG1478 treatment (Figure 1C). Western blot analysis indicated significant reduction in the expression of pro-fibrotic gene TGF-β1 and collagen IV in diabetic kidneys (Figure 1D). Real-time qPCR assay also exhibited a remarkable increase in pro-fibrotic genes including collagen-IV and TGF-β1 (Figure 1E and 1F). AG1478 treatment also significantly decreased renal injury and fibrosis markers (Figure 1A–1F). As shown in Figure 2A, apoptosis cells stained in bright green by TUNEL assay were remarkably increased in diabetic mice. AG1478 treated group significantly reversed diabetes-induced nephrocyte apoptosis. The expression of pro-apoptotic protein Bax in mouse kidneys was assessed by western blot analysis. As expected, the results indicated that treatment with AG1478 inhibited diabetes-induced Bax upregulation (Figure 2B).

### AG1478 attenuated renal EGFR signaling activation in diabetic mice

**Figure 3:**
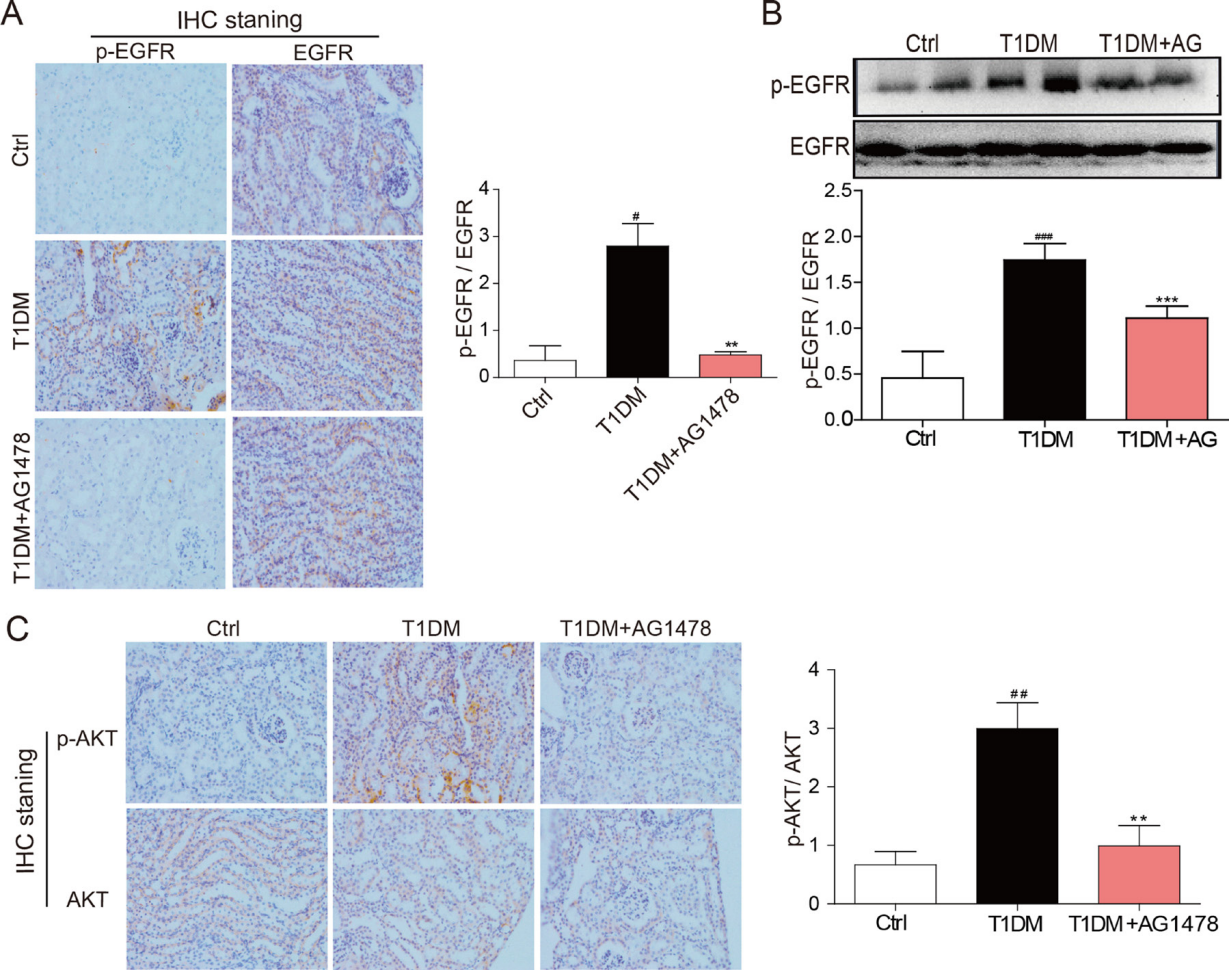
AG1478 attenuate diabetes-induced EGFR signaling activation in diabetic kidney (**A**) Representative images for the histochemical staining for p-EGFR and EGFR expression in the formalin-fixed renal tissues (200× magnification). (**B**) Western blot analysis for the expression of p-EGFR in renal tissue. And statistic figure was shown, data were presented as mean ± SDs. (**C**) Representative images for the histochemical staining for p-AKT and AKT expression in the formalin-fixed renal tissues (200× magnification). (Eight mice in each group were used for above analysis. **P < 0.01, ***P < 0.001 versus DN, ^#^*P* < 0.05, ^##^*P* < 0.01, ^###^*P* < 0.001 versus vehicle control (Ctrl)).

The EGFR signaling is activated in early diabetes and plays an important role in kidney hypertrophy and fibrosis. Here we observed that EGFR phosphorylation was up-regulated in diabetic mice, both at cellular levels and total protein levels (Figure 3A, 3B). However, AG1478 treatment dramatically decreased EGFR phosphorylation in diabetic kidneys (Figure 3A, 3B), suggesting that AG1478 eliminated EGFR activation. Since EGFR is known to turn on PI3K/AKT signaling [16], we explored whether AG1478 regulates this major downstream target phosphorylation. We found that AKT was also significantly activated in diabetic kidneys, which was markedly inhibited in AG1478-treated animals (Figure 3C). These data suggested that EGFR and AKT were activated during DN progression, and AKT phosphorylation directly responded to EGFR activation.

### AG1478 attenuated diabetes-induced renal oxidative stress and ER stress

**Figure 4:**
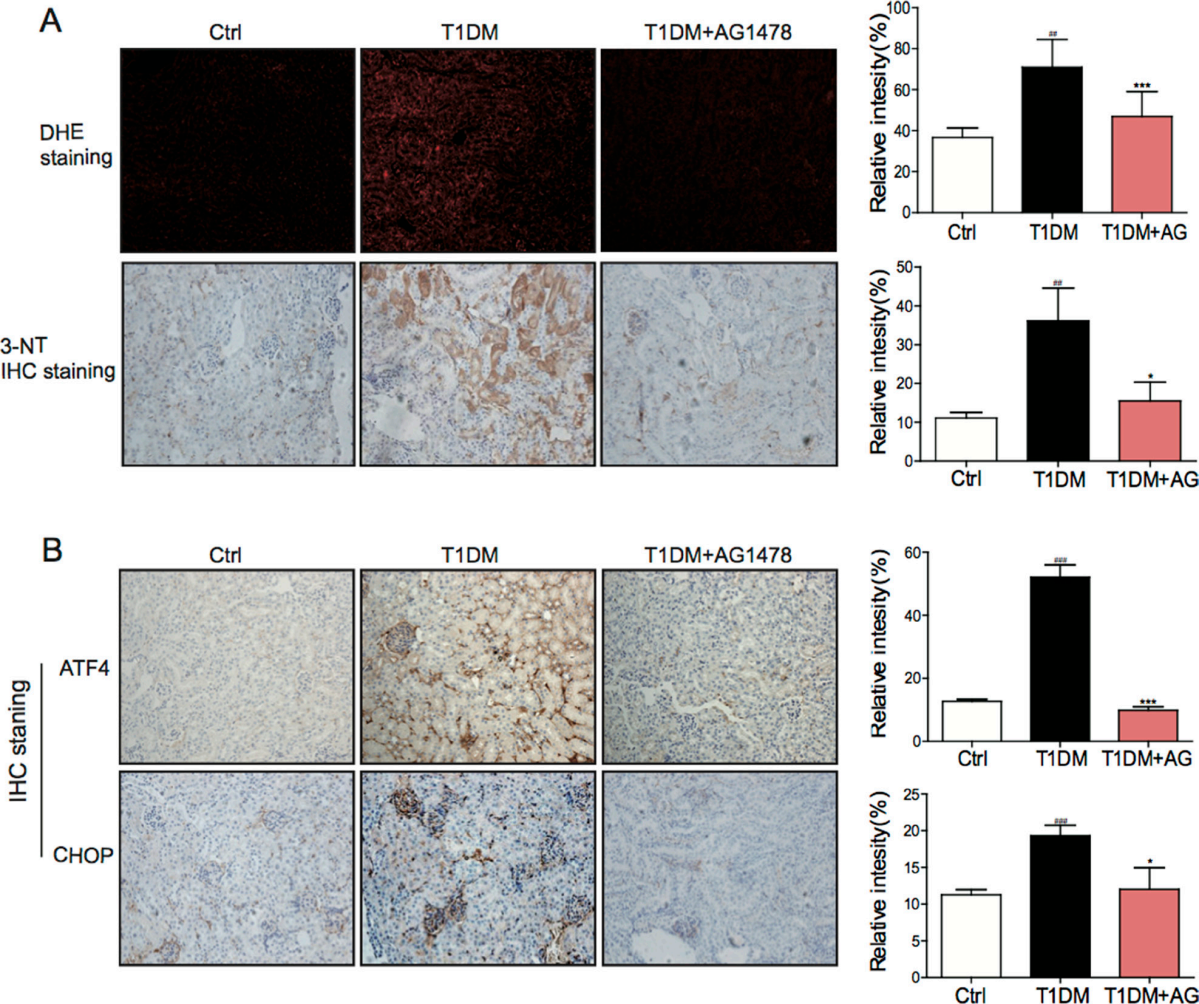
AG1478 attenuate diabetes-induced oxidative stress and endoplasmic reticulum stress (**A**) Representative images for DHE staining using the formalin-fixed renal tissues as described in materials and method (200× magnification). Representative images for immunohistochemial staining of 3-NT accumulation using the formalin-fixed renal tissues as described in materials and methods section(200× magnification). And statistic figure was shown, data were presented as mean ± SDs. (**B**) Representative images for immunohistochemical staining of ATF4 and CHOP accumulation using the formalin-fixed renal tissues as described in Materials and methods (200× magnification). And statistic figure was shown, data were presented as mean ± SDs. (Eight mice in each group were used for above analysis. *P < 0.05, ***P < 0.001 versus DN; ^##^*P* < 0.01, ^###^*P* < 0.001 versus vehicle control (Ctrl)).

Mounting evidence has established that oxidative stress and ER stress are entwined phenomena, contributing to the diabetes-induced pathological changes. Therefore, we investigated whether oxidative stress and ER stress were involved in the attenuation of diabetic nephropathy after EGFR inhibition. IHC staining analysis showed that both oxidative stress markers (DHE and 3-NT) and ER stress markers (ATF4 and CHOP) were increased in STZ-induced diabetic kidneys (Figure 4A, 4B). Significantly, AG1478 administration could eliminate these changes. The results indicated that AG1478 treatment markedly reduced renal oxidative stress (Figure 4A) and inhibited renal ER stress (Figure 4B), suggesting that the protective effects of EGFR blockade may be associated with the inhibition of oxidative stress and ER stress.

### AG1478 and NAC inhibited HG-induced ROS generation, ER stress, apoptosis, and fibrosis

**Figure 5:**
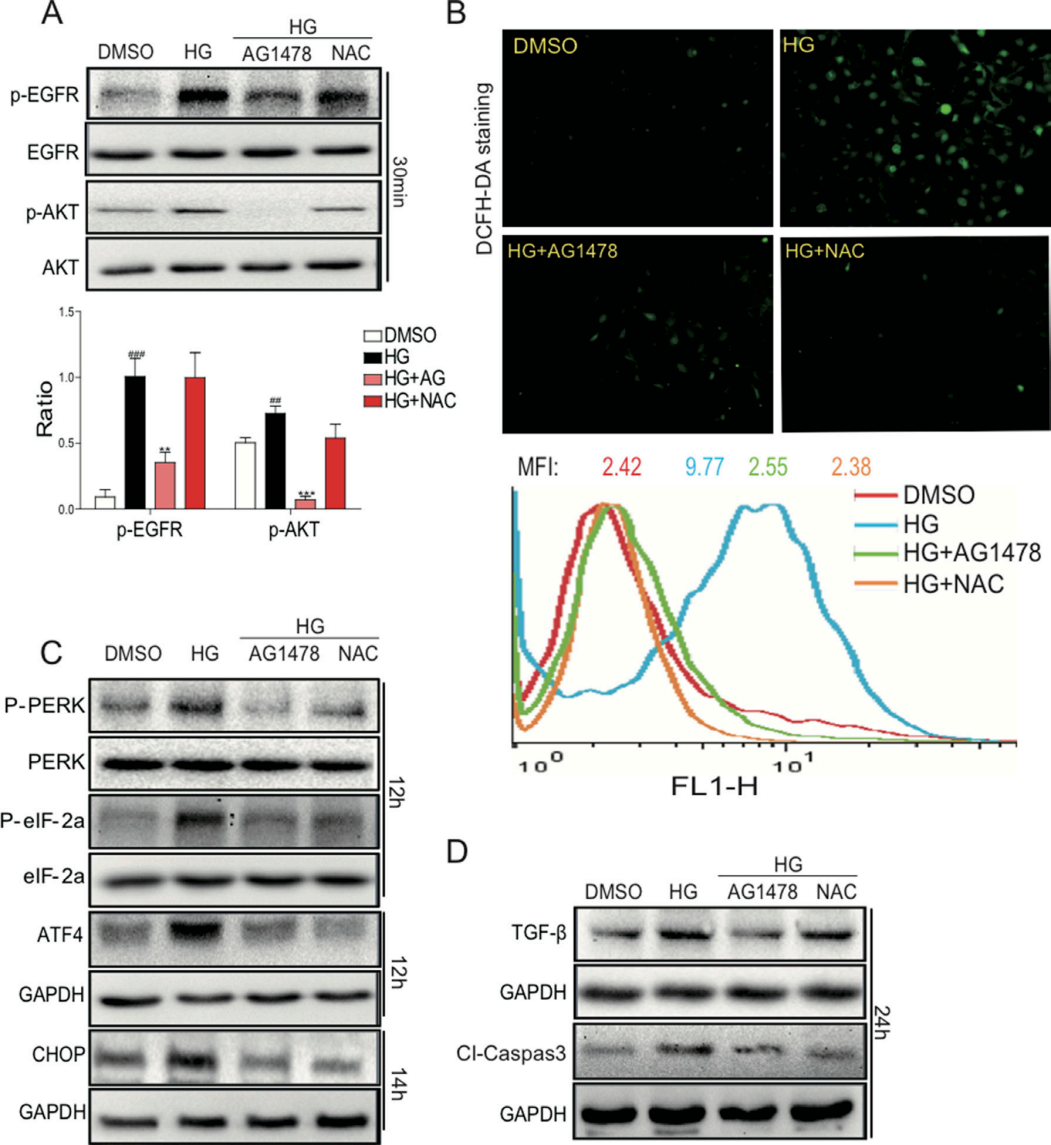
AG1478 and NAC attenuate HG-induced EGFR signaling activation, ROS generation, endoplasmic reticulum stress, cell firosis and apoptosis in SV40 cells SV40 cells pretreated with AG1478 (10 μM) or NAC (10 mM) for 1 h were incubated with HG (33 mM) for 1 h. Then cells were lysed and the extracted total proteins were processed for the detection of p-EGFR and p-AKT using Western blot; and statistic figure was shown, data were presented as mean ± SDs. (Six mice in each group were used for above analysis (**A**). (**B**) AG1478 and NAC inhibit high glucose-induced ROS generation. SV40 cells pretreated with AG1478 (10 μM) or NAC (10mM) for 1h were incubated with HG (33 mM) for 2h. DCFH-DA probes were loaded and the ROS positive cells were detected using the fluorescence microscope. Also, after loading with the probes, cells were processed to flow cytometry analysis for O2 level, and mean fluorescence intensity (MFI) value was determined. Data were presented as mean ± SDs. (*n* = 3 for each experiment. **P < 0.01, ***P < 0.001 versus HG; ^##^*P* < 0.01, ^###^*P* < 0.001 versus DMSO). (**C**) Western blot analysis for the protein expression of ERS-related proteins ATF4 and CHOP in SV40 cells. (**D**) Western blot analysis for the fibrosis and apoptosis protein expression of TGF-β1 and Cleaved-Caspase 3 in SV40 cells.

*In vivo* study has showed that EGFR inhibitor AG1478 can attenuate renal oxidative stress and renal injury in mouse model with type 1 diabetes. An antioxidant, N-acetyl-L-cysteine (NAC) which is well-known to mitigate the increased oxidative stress, was used for *in vitro* study. To investigate whether the inhibition of EGFR activity and ROS affects the aforementioned damage *in vitro*, mesangial cells cultured in high-glucose medium were pretreated with AG1478 or NAC. As indicated in Figure 5A, renal mesangial SV40 cells endogenously expressed a relative high amount of EGFR and AKT. HG treatment significantly increased the phosphorylation of both EGFR and AKT in SV40 cells, whereas these alterations were reversed by AG1478 pre-treatment. In contrast, NAC pre-treatment had no obvious effects.

Furthermore, we determined the effects of EGFR inhibitor on HG-induced ROS generation and cell damage. As shown in Figure 5B, HG treatment for 1h markedly increased ROS generation, which was significantly reduced by pre-treatment with either AG1478 or NAC in SV40 cells, as indicated by DCFH-DA fluorescence staining and flow cytometry. These results indicated that EGFR signaling may be a mediator of HG-induced ROS generation. We further found that AG1478 or NAC administration markedly reduced HG-induced ATF4/CHOP expression and PERK/eIF2α phosphorylation in SV40 cells (Figure 5C). We next examined the effects of EGFR inhibitor or ROS scavenger on the renal cell fibrosis and apoptosis in HG-treated SV40 cells (Figure 5D). Elevated protein expression of TGF-β1 and Caspase-3 by HG could be reduced by either AG1478 or NAC. The results indicated that HG could induce the DN phenotypes in SV40 cells via EGFR activation, both EGFR inhibitor and ROS scavenger could prevent the cell damages, suggesting that EGFR and ROS play pivot roles in mediating hyperglycemia-induced renal pathogenesis.

### EGFR and AKT siRNA inhibited HG-induced markers for ER stress, apoptosis, and fibrosis in SV40 cells

**Figure 6:**
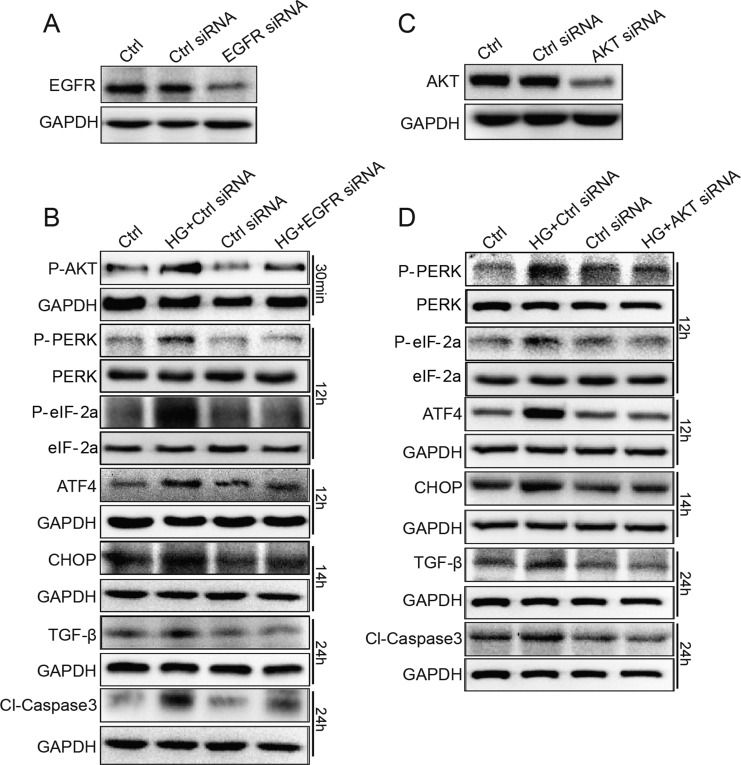
EGFR and AKT mediate HG-induced ER stress and cell damage in SV40 MES 13 cells (**A**) SV40 cells were transfected with EGFR siRNA or control siRNA for 48 h, the expression of EGFR was detected by Western blot analysis. (**B**) EGFR silencing by siRNA reduced HG-induced ER stress, fibrosis and apoptosis. (**C**) SV40 cells were transfected with AKT siRNA or control siRNA for 48 h, the expression of AKT was detected by Western blot analysis. (**D**) AKT silencing by siRNA reduced HG-induced ER stress, fibrosis and apoptosis. Data were obtained from three independent experiments.

In order to avoid the non-specificity of small-molecular inhibitor AG1478, we used EGFR and AKT siRNA to specifically silence EGFR/AKT expression in SV40 cells. As indicated in Figure 6A, western blot analysis demonstrated that the transfection of EGFR siRNA resulted in a remarkable decrease in EGFR expression in SV40 cells, compared to control scrambled siRNA. Furthermore, as shown in Figure 6B, EGFR siRNA-transfected group markedly inhibited HG-induced AKT phosphorylation, ATF4 and CHOP protein expression, PERK and eIF2α phosphorylation, TGF-β1 protein expression, and Caspase-3 activation. Figure 6C and 6D shows that inhibition of AKT suppression by siRNA also decreased HG-induced PERK/eIF2α phosphorylation and ATF4/CHOP/TGF-β1/Caspase-3 protein expression in SV40 cells. Directly knocking down EGFR using siRNA significantly inhibited the phosphorylation of AKT induced by HG (Figure 6B), confirming that AKT is a downstream mediator of EGFR. In conclusion, knocking down EGFR and AKT prevented the HG-induced ER stress, pro-apoptosis, and fibrosis, indicating that the EGFR/AKT/ROS/ER stress pathway contributes to the hyperglycemia-induced renal cell damages.

## DISCUSSION

**Figure 7:**
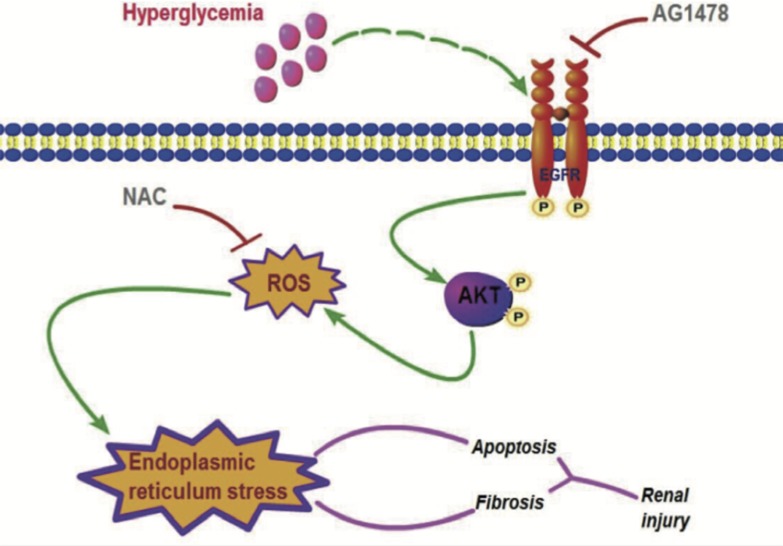
Scheme for EGFR/AKT/ROS/ER stress signaling pathway in preventing DN Inhibition of EGFR by AG1478 eliminated AKT phosphorylation, sequentially reduced oxidative stress and ER stress, decreased diabetes-induced renal.

Our studies demonstrated that renal EGFR phosphorylation stimulates ROS accumulation and ER stress in STZ-induced diabetic animals. Administration of EGFR inhibitor AG1478 attenuated DN, accompanied with EGFR/AKT inactivation and the decreased ROS and inhibited ER stress. Then, we use *in vitro* study to confirm this pathway in renal mesangial cells. AG1478 blocked HG-induced AKT phosphorylation, ROS production, and ER stress, while NAC only blocked ROS production and ER stress. To avoid the specificity of AG1478 and to see the role of AKT, we used specific siRNA to silence EGFR and AKT, and similar results were observed. Finally, a clear pathway “from EGFR to AKT, ROS, and ER stress, and finally to kidney fibrosis” has been demonstrated (Figure 7). These results suggested that targeting EGFR could be future therapeutic approach for DN to reduce the physical and psychosocial burdens of diabetes.

We previously discovered that EGFR has a key role in the pathogenesis cardiac damage and remodeling in STZ induced diabetic animals [9]. EGFR activation medicates renal repair after acute injury [17]. However, further exploration illustrated that persistent EGFR activation is an essential step in renal fibrosis induced by unilateral ureteral obstruction [18], subtotal nephrectomy [19], renal hypertension [20], or angiotensin II/endothelin [21, 22] triggered renal injury. Here we provided direct evidence that suppressing EGFR prevents renal fibrosis and apoptosis in mice. These data are consistent with increased EGFR signaling leads to pathological damage in DN patients [23]. We also found that AKT knockdown also inhibited TGF-β1 expression in renal mesangial SV40 cells, which is consistent with our previous observations in cardiomyocytes [9, 24] and kidney epithelial cells [25]. TGF-β1 is thought to be the core factor that contributes to the renal fibrosis of DN. Recently, experimental evidence showed that TGF-β1 participates in the activation of the PI3K/AKT pathway, suggesting that AKT is a downstream mediator of TGF-β1 [26, 27]. Thus, TGF-β and AKT may positively crosstalk to regulate each other, promoting tissue fibrosis and other phenotypes.

Accumulating evidence indicates that oxidative stress and endoplasmic reticulum stress contribute to development and progression of diabetes and complications. Both increased ROS production and ER stress were observed in the current study, with similar results with other independent groups [28, 29]. In this study, we set to explore the molecular mechanisms underlying ROS/ER stressduring DN progression. As a direct target of EGFR, AKT phosphorylation is blocked by EGFR inhibition through either AG1478 or EGFR siRNA, and the phosphorylation of AKT regulates oxidative stress and ER stress in DN. Activated EGFR was reported to induce NOX activation via ERK signaling pathway, and consequently influenced the production of ROS [17, 30]. However, we found that EGFR directly caused AKT phosphorylation. Evidence has demonstrated that AKT activation positively regulates ER stress via PrPC and Akt-MnSOD pathway in mesenchymal stem cells [31]. Also, PI3K-Akt signal inhibition affects expression of genes related to endoplasmic reticulum stress in primary goose hepatocytes [32]. Thus, our finding that AKT knockdown prevents HG-induced protein expression involved in ER stress activation further confirmed the regulation of AKT on ER stress in renal cells. Interestingly, NAC, a scavenger of ROS, could also inhibit ER stress, suggesting that ROS accumulation could trigger ER stress. ROS is linked to ER stress in other systems, such as rat cardiac cells, and human hepatoma cells [33, 34]. Reports on the interactions between ROS and ER stress are still controversial and not clear [9, 35, 36]. Here we provide direct evidence that inhibition of ROS could alleviate ER stress in SV40 cells.

It is known that EGFR activation is a key component in diabetic damage; it involves in insulin sensitivity [37], and leads to diabetic cardiomyopathy [9]. Chen et al. reported Hippo pathway may be linked to EGFR-mediated renal epithelial injury in diabetes, however, they failed to include oxidative stress and ER stress pathway as one of the critical contributors to these pathological changes [38]. Oxidative stress is well characterized in cardiovascular disease, and ROS trans-activates EGFR through HB-EGF production [39]. Our study confirmed the crosstalk between ROS accumulation and EGFR transactivation in renal system, which has been reported to mediate various cellular responses in inflammation, fibrosis, and apoptosis [40, 41].

Using knock-down approach to inhibit EGFR and AKT, we successfully rescued ROS/ ER stress mediated apoptosis and fibrosis, further illustrated that EGFR/AKT pathway plays a pivot role in the progress of DN. Future investigation will focus on confirming these molecular cascades in living animals. We expect that renal microvascular function is altered, as Akhtar's group showed that in vascular smooth muscle cells (VSMCs), inhibition of EGFR/ErbB2 reverses the vascular dysfunction. EGFR could be a potential clinical strategy in diabetic patients with microvascular complications [42]. Since EGFR signaling has various roles in cardiovascular system [43], controlling EGFR phosphorylation is important to improve the prognosis of diabetic patients.

Due to the pathogenic effects of EGFR in multiple studies, EGFR antagonism by antibodies or small-molecule inhibitors has been shown to have beneficial effects in cancer patients [44]. EGFR monoclonal antibodies are tumor specific and react with breast and lung carcinomas and malignant gliomas [45]. EGFR antagonism using AG1478 reduces cardiac fibrosis in diabetic mice and improved metabolic status via inhibition of eIF2α/ATF4 pathway [46]. Inhibition of EGFR using AG1478 is also reported to prevent progressive kidney injury through mTOR/AMPK pathway. Here we have utilized STZ-induced diabetic mice model and demonstrate that AG1478 markedly attenuates ER stress and promotes renal fibrosis through inhibition of EGFR/AKT activation.

In conclusion, we demonstrated that EGFR plays a pivot role in diabetic nephropathy pathogenesis via up-regulating ROS generation and ER stress. Inhibition of EGFR attenuates damage of DN through EGFR/AKT/ROS/ER stress signaling, which could be a potential therapeutic target in diabetic kidney diseases.

## MATERIALS AND METHODS

### Reagents, cell culture and treatment

AG1478 and NAC were purchased from Sigma (St.Louis,MO). AG1478 was dissolved in DMSO for *in vitro* experiments and in sodium carboxyl methyl cellulose (CMC-Na) (0.5%) for *in vivo* experiments. NAC was dissolved in DMSO for *in vitro* experiments. SV40 MES 13 mesangial cell line was obtained from the Shanghai Institute of Biochemistry and Cell Biology (Shanghai, China) and cultured in 3:1 mixture of DMEM medium (Gibco, Eggenstein, Germany) containing 5.5 mmol/L of D-glucose and Ham's F12 medium with 14mM HEPES supplemented with 10% FBS, 100U/ml of penicillin, and 100 mg/mL of streptomycin. In the high glucose-treated group (HG), cells were incubated with a DMEM medium containing 33mmol/L of D-glucose.

### Animals

The animals were obtained from Animal Center of Wenzhou Medical University. Protocols used for all animal studies were approved by the Wenzhou Medical University Animal Policy and Welfare Committee. All mice had free access to food and water at all times. Diabetes mellitus was induced in male C57/BL6 mice 8 to 12 weeks old, weighing 18 to 22g by a single intraperitoneal (i.p.) injection of STZ (Sigma Chemicals, St. Louis, MO) at the dose of 100 mg/kg dissolved in 100 mM citrate buffer (pH 4.5). Control animals received buffered saline alone. One week after STZ injection, blood glucose levels were measured using a Glucometer by mandibular vein puncture blood sampling. Mice with fasting-blood glucose > 12 mmol/L were considered diabetic and were used for the further study. The compound treatment was started 1 week after STZ injection. Diabetic mice were orally treated with AG1478 (AG, 20 mg/kg), vehicle (CMC-Na) by gavage every other day for 8 weeks (*n* = 8 in each group). The corresponding control groups were treated with vehicle for 8 weeks (*n* = 8). At the indicated time points, blood glucose was measured and body weight was recorded (Supplementary Figure 1). 56 days after the first treatment, mice were sacrificed under anestheisa. The body weight was recorded at the end of death and the blood samples were collected and centrifuged at 4°C at 3000 rpm for 10 min to collect the sera. Kidney tissues were collected and embedded in 4% paraformaldehyde for pathological analysis and/or snap-frozen in liquid nitrogen for gene and protein expression analysis.

### Western blot analysis

Cell and tissue homogenates were prepared. Protein samples (30–80 μg) were subjected to 10% sodium dodecyl sulfate-polyacrylamide gel electrophoresis, and transferred onto polyvinyldene fluoride membrane (Bio-Rad Laboratory, Hercules, CA). After incubating in blocking buffer (5% milk in tris-buffered saline containing 0.05% Tween 20) for 1.5 h at room temperature, membranes were incubated with different primary antibodies overnight at 4°C. Antibodies for p-EGFR/EGFR, p-AKT/AKT, p-PERK/PERK, p-eIF2α/ eIF2α, Bax, GAPDH, TGF-β1, collagen IV, CHOP, cleaved-Caspase 3 and were obtained from Santa Cruz Technology (Santa Cruz, CA), and antibodies for ATF4, CHOP were purchased from Abcam (Cambride, MA). Then membranes were washed in TBST and reacted with secondary horseradish peroxidase-conjugated antibody (Santa Cruz, CA; 1:5000) for 1–2 h at room temperature. Antigen-antibody complexes were then visualized using enhanced chemiluminescence reagents (Bio-Rad, Hercules, CA). The density of the immunoreactive bands was analyzed using Image J software (NIH, Bethesda, MD).

### Reverse transcription and real-time quantitative PCR

Total RNA was isolated from cells and tissues (50–100 mg) using TRIZOL (Invitrogen, Carlsbad, CA) according to the manufacturer's instructions. Reverse transcription and quantitative PCR were performed using M-MLV Platinum RT-qPCR Kit (Invitrogen, Carlsbad, CA). Real-time qPCR was carried out using the Eppendorf Real plex 4 instrument (Eppendorf, Hamburg, Germany). The following primers were synthesized from Invitrogen: collagen IV, sense: TGGCCTTGGAGGAAACTTTG, and antisense: CTTGGAAACCTTGTGGACCAG; TGF-β1, sense: TGACGTCACTGGAGTTGTACGG, and antisense: GGTTCATGTCATGGATGGTGC; and b-actin, sense: CCGTGAAAAGATGACCCAGA, and antisense: TACGACCAGAGGCATACAG. The relative amount of each gene was normalized to the amount of β-actin.

### Determination of ROS generation by fluorescent microscope and flow cytometry

In order to analyze the ROS generation, a subtype of ROS such as hydrogen peroxide (H2O2) was detected using 2 μM DCFH-DA, respectively, as described previously [47]. The fluorescence intensity for 10,000 events was acquired using FACS, and cellular images were captured under the Nikon fluorescence microscope (400×amplification; Nikon, Japan).

### Transient transfection of EGFR and AKT si-RNA

The small interfering RNA (si-RNA) specifically targeting the nucleotides of EGFR or AKT and its control si-RNA containing negative scrambled sequences were obtained from Gene Pharma LTD. (Shanghai, China). Transfection of SV40 cells with siRNAs was carried out using LipofectAMINE^TM^ 2000 (Invitrogen, Carlsbad, CA), according to the manufacturer's instruction. After 48h of transfection, the transfected cells were then treated with HG for the following experiments.

### Determination of renal superoxide production

We evaluated renal superoxide production with *in situ* dihydroethidium (DHE) staining using the method described previously [47]. In brief, kidneys from mice were excised, immediately embedded in OCT compound, and cut into 5 μm-thick sections. The sections were incubated with DHE in PBS (10 mmol/L) in a dark and humidified container at 37°C for 45 min. DHE is oxidized upon reaction with superoxide to ethidium bromide, which binds to DNA in the nucleus and fluoresces red. The images were viewed under the fluorescence microscope (λex 490 nm, λem610 nm, 400×amplification; Nikon, Japan).

### Measurement of Serum Biomarkers

The components of serum albumin were detected using commercial kits (Nanjing Jiancheng Bioengineering Institute, Jiangsu, People's Republic of China).

### Histological analysis

Kidneys were fixed in 4% paraformaldehyde solution, embedded in paraffin, and sectioned at 5 μm. After dehydration, sections were stained with hematoxylin and eosin (H&E), masson's trichrome, sirius red, respectively, according to the previously reported methods [48]. The stained sections were then viewed under the fluorescence microscope (400×amplification; Nikon, Japan).

### Immunohistochemistry analysis

Kidneys were fixed in 4% paraformaldehyde solution, embedded in paraffin, and sectioned at 5μm. After dehydration, sections were subjected to antigen retrieval in 0.01 mol/L citrate buffer (pH 6.0) by microwaving, and then placed in 3% hydrogen peroxide in methanol for 30 min at room temperature. After blocking with 5% BSA, the sections were incubated with anti-p-EGFR antibody (1:300, Santa Cruz, CA, USA), anti-p-AKT antibody (1:300, Santa Cruz, CA, USA), anti-3-NT antibody (1:500, Abcam Inc, MA), anti-ATF-4 antibody (1:500, Abcam Inc, MA), or anti-CHOP antibody(1:500, Abcam Inc, MA), respectively, overnight ant 4°C, followed by the appropriate secondary antibody (1:200, Santa Cruz, CA, USA). The reaction was visualized with DAB solution. After counterstaining with hematoxylin, the sections were dehydrated and viewed under a light microscope (400×amplification; Nikon, Japan).

### TUNEL staining

Kidney tissue sections of 5 μm were used for the terminal deoxynucleotidyl transferase-mediated dUTP nick end labeling (TUNEL) apoptosis detection kit (R&D Systems, Minneapolis, MN) according to the manufacturer's instruction. TUNEL positive cells were imaged under a fluorescence microscope (400×amplification; Nikon Tokyo, Japan).

### Statistical analysis

Results are expressed as mean ± SDs of three independent experiments for the *in vitro* studies or of 7 mice for the *in vivo* experiments. The statistical significance of differences between groups was obtained by the student's *t*-test or ANOVA multiple comparisons in GraphPad Pro5.0 (Graphpad, San Diego, CA). Differences were considered to be significant at *P < 0.05; **P < 0.01; ***P < 0.005.

## SUPPLEMENTARY MATERIALS FIGURES


