# Supplementary material for: EGFR inhibition attenuates diabetic nephropathy through decreasing ROS and endoplasmic reticulum stress
Source: Oncotarget. 2017 Mar 6;8(20):32655–67. doi: 10.18632/oncotarget.15948 (PMC5464817; doi:10.18632/oncotarget.15948)
Supplement: Supplementary file 1 [file oncotarget-08-32655-s001.pdf]

EGFR inhibition attenuates diabetic nephropathy through decreasing ROS and endoplasmic reticulum stress

Supplementary Materials

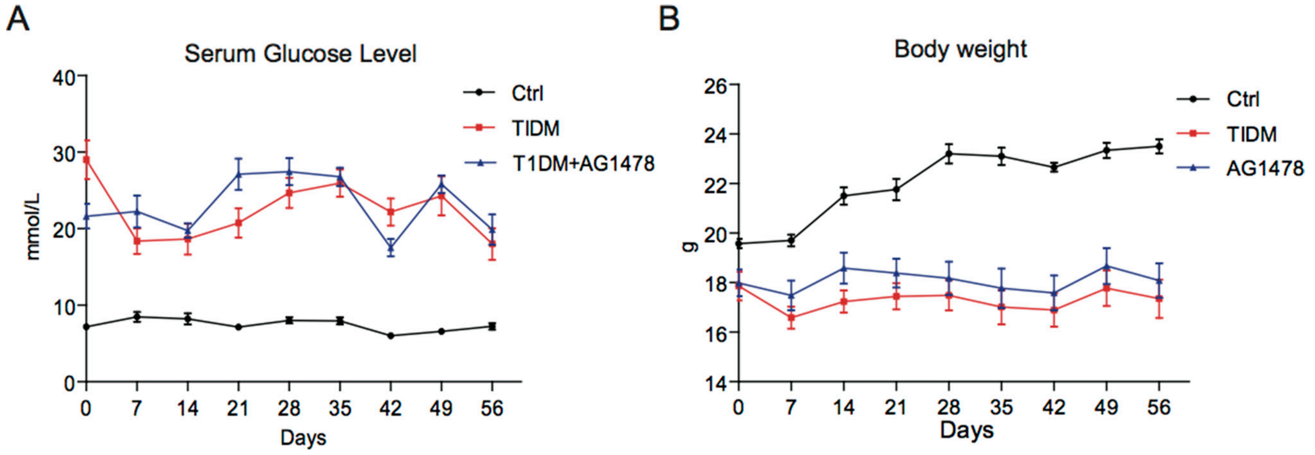

Supplementary Figure 1: AG1478 administration did not affect blood glucose level and body weight. STZ-induced diabetic mice showed elevated blood glucose level (**A**) and the relatively body weight (**B**) during the animal experiment interval. (Eight mice in each group were used for above analysis. Data were expressed as mean ± SDs).
